# Distribution of sputum cellular phenotype in a large asthma cohort: predicting factors for eosinophilic vs neutrophilic inflammation

**DOI:** 10.1186/1471-2466-13-11

**Published:** 2013-02-26

**Authors:** Florence N Schleich, Maité Manise, Jocelyne Sele, Monique Henket, Laurence Seidel, Renaud Louis

**Affiliations:** 1 Department of Respiratory Medicine, CHU Sart-Tilman B35, Liege 4000, Belgium; 2 Medical Informatics and Biostatistics, University of Liege, Liege, Belgium

**Keywords:** Asthma, Induced sputum, Eosinophil, Neutrophil

## Abstract

**Background:**

Phenotyping asthma according to airway inflammation allows identification of responders to targeted therapy. Induced sputum is technically demanding. We aimed to identify predictors of sputum inflammatory phenotypes according to easily available clinical characteristics.

**Methods:**

This retrospective study was conducted in 508 asthmatics with successful sputum induction recruited from the University Asthma Clinic of Liege. Receiver-operating characteristic (ROC) curve and multiple logistic regression analysis were used to assess the relationship between sputum eosinophil or neutrophil count and a set of covariates. Equations predicting sputum eosinophils and neutrophils were then validated in an independent group of asthmatics.

**Results:**

Eosinophilic (≥3%) and neutrophilic (≥76%) airway inflammation were observed in 46% and 18% of patients respectively. Predictors of sputum eosinophilia ≥3% were high blood eosinophils, FE_NO_ and IgE level and low FEV_1_/FVC. The derived equation was validated with a Cohen’s kappa coefficient of 0.59 (p < 0.0001). ROC curves showed a cut-off value of 220/mm^3^ (AUC = 0.79, p < 0.0001) or 3% (AUC = 0.81, p < 0.0001) for blood eosinophils to identify sputum eosinophilia ≥3%. Independent predictors of sputum neutrophilia were advanced age and high FRC but not blood neutrophil count.

**Conclusion:**

Eosinophilic and paucigranulocytic asthma are the dominant inflammatory phenotypes. Blood eosinophils provide a practical alternative to predict sputum eosinophilia but sputum neutrophil count is poorly related to blood neutrophils.

## Background

Asthma is a heterogeneous disease of the airways. The traditional guidelines for asthma diagnosis include suggestive clinical symptoms and the demonstration of airflow variability. However, symptoms and lung function are insensitive in reflecting the underlying airway inflammation. There is increasing evidence that phenotyping asthma according to airway inflammation can allow the identification of subgroups of patients who are more likely to respond to targeted therapy. In particular, important studies have confirmed that eosinophilic airway inflammation most reliably predicts the response to anti-inflammatory treatment such as inhaled corticosteroid [1,2] and anti-IL5 [3,4]. Recent studies have demonstrated the usefulness of induced sputum to guide asthma treatment [5,6]. These studies showed that normalizing airway eosinophilic inflammation allowed better control of asthma with reduced exacerbations and hospital admissions. There is however no evidence that inhaled corticosteroids may improve short term asthma control in the absence of uncontrolled eosinophilic inflammation as encountered in pauci-granulocytic asthma [7]. On the other hand data suggest that neutrophilic asthma could be best targeted by using clarithromycin [8]. Characterising the inflammatory phenotype in patients with chronic respiratory symptoms can thus be more important than giving an “asthma” label to predict response to anti-inflammatory treatment. The technique of induced sputum that allows non-invasive collection of airway cells is considered as the gold standard to identify inflammatory asthma phenotype. It is however technically demanding and time consuming. Exhaled nitric oxide (FE_NO_) has already been identified [9,10] as a valid marker of airway eosinophilic inflammation. We currently however lack of marker predicting airway neutrophilic inflammation. It is unclear whether systemic inflammation is able to predict inflammatory phenotypes either eosinophilic or neutrophilic. This alternative test is biologically plausible since the infiltrating granulocytes in the airway are bone marrow-derived cells which access the airway through the circulation. The appeal of the approach comes from the ease of sample collection of peripheral blood from subjects of all ages and clinical characteristics.

The purpose of this study was twofold. In a large asthmatic population encompassing the all disease severity spectrum, we first sought to assess the proportion of asthmatic patients displaying eosinophilic vs neutrophilic vs paucigranulocytic phenotypes based on sputum cell analysis. Secondly we aimed at determining factors associated with eosinophilic and neutrophilic phenotypes.

## Methods

### Subject characteristics and study design

We conducted a retrospective study on a series of 508 patients with asthma recruited from the University Asthma Clinic of Liege between 1 October 2005 and 27 June 2011 and who had a successful sputum induction. Their demographic and functional characteristics are summarised in Table 1. Patients underwent FE_NO_ measurement at a flow rate of 50 ml/s according to the ERS/ATS recommendations (NIOX, Aerocrine, Sweden). FE_NO_ was first measured and followed by spirometry with bronchodilation, sputum induction and blood sampling. All tests were performed on the same day. Asthma was diagnosed based on the presence of chronic respiratory symptoms such as cough, breathlessness or dyspnoea together with the demonstration of airflow variability. The latter was defined by airway hyper-responsiveness shown by one or more of the following: increase in Forced expiratory volume in 1 s (FEV_1_) of >12% and 200 ml following inhalation of 400 μg salbutamol or inhaled concentration of methacholine provoking a 20% fall in FEV_1_ of <16 mg/ml. Methacholine challenge was performed according to a standardised methodology as previously described [11]. Subjects were characterised as atopic if they had at least one positive specific IgE (>0.35 kU/l; Phadia) for at least one common aeroallergen (cat, dog, house dust mites, grass pollen, tree pollen and a mixture of moulds). Quality of life was assessed using the self-administered Asthma Quality of Life Questionnaire (AQLQ) [12] and asthma control by the Juniper Asthma Control Questionnaire (ACQ) [13]. Sputum was induced and processed as previously reported [14] and was successful in 78% of the patients encountered in our asthma clinic (data not shown). Cell count were estimated on samples centrifuged (Cytospin) and stained with Diff Quick after counting 500 cells (Dade, Brussels, Belgium). Eosinophilic phenotype was defined as ≥3% sputum eosinophil count while neutrophilic phenotype consisted of ≥76% sputum neutrophil counts [15]. When deriving the upper limit of the 90% reference interval, abnormally high neutrophil count ranges from 49% according to Spanevello [16] to 93% according to Thomas [17], most of the authors setting the threshold between 61% and 76%. We defined an abnormally high sputum neutrophil count as a percentage ≥76% (>mean + 1.7SD of our reference value found in healthy subjects matched for age (34.9+/-24.3%; n = 113)) [15].

**Table 1:** Demographic, functional and inflammatory characteristics for the whole population

**Characteristics**	
N.	508
Sex (M/F)	201/307
Age, yrs	52 (19–88)
Height, cm	167 ± 9
Weight, kg	74 ± 16
Atopy (Y/N)	296/212 (58%)
Current Smoker (n) (pack-yr)	101 (22 (0.5-60) pack-yr)
Ex-smokers (n) (pack-yr)	99 (15 (0.5-90) pack-yr)
FEV_1_,% predicted	84 ± 19
Sputum eosinophils,%	2 (0–94)
Sputum neutrophils,%	45 (0–100)
ICS therapy	
Steroid naïve	153 (30%)
Low dose ICS	73 (15%)
Moderate dose ICS	138 (27%)
High dose ICS	144 (28%)

Data are presented as mean ± SD or median (range). *FEV*_*1*_, forced expiratory volume in 1 s. *ICS*: inhaled corticosteroids. Low dose: ≤500 μg/day beclomethasone; Moderate dose: 500-1000 μg/day beclomethasone; High dose: >1000 μg/day beclomethasone.

### Statistical analyses

The results were expressed as mean ± SD for continuous variables; median and interquartile ranges (IQR) were preferred for skewed distributions. For categorical variables, the number of observations and percentages were given in each category. Comparisons between different subgroups were performed with a Kruskal-Wallis test. The Spearman correlation coefficient was used to measure the association between clinical parameters. The receiver-operating characteristic (ROC) curve was constructed to determine the concentration of blood eosinophils or neutrophils which best identified a sputum eosinophil count ≥3% or a sputum neutrophil count ≥76% respectively. Logistic regression analysis was used to assess the relationship between the binary outcome (sputum eosinophil count ≥3% or sputum neutrophil count ≥76%) and a set of covariates, individually or in combination (gender, age, height, weight, atopy, smoking status, IgE, blood eosinophil and neutrophil count, FEV_1_%, FEV_1_/FVC, TLC, FRC, KCO, PC_20_M, Reversibility, Fibrinogen, CRP, FE_NO_, ACQ, AQLQ, ICS therapy). The results were considered to be significant at the 5% critical level (p < 0.05). The ability of the equation to predict sputum eosinophilia or neutrophilia was tested in an independent population of 178 asthmatics recruited between July 2011 and May 2012. The demographic, functional and inflammatory characteristics of the validation population were similar to those of the study population.

As IgE levels were missing in 40 patients, the equation predicting sputum eosinophilia was validated in 138 patients. The agreement between predicted and observed value was tested by Cohen Kappa’s coefficient. Calculations were done using SAS version 9.1 (SAS Institute, Cary, North Carolina, USA).

This study was conducted with the approval of the ethics committee of CHU Liege B70720096732, reference Liege 2009/161.

## Results

The demographic and functional characteristic of the patients are given in Table 1.

**Figure 1:**
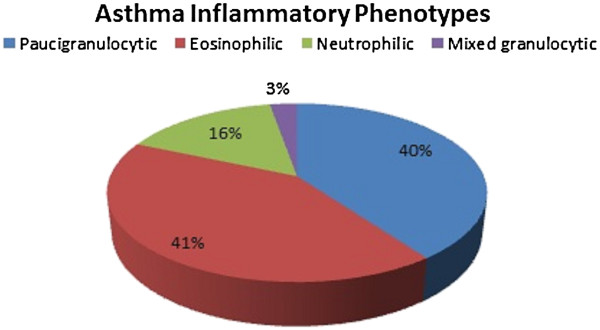
Proportion of various inflammatory phenotypes according to cellularity of induced sputum in a large cohort of asthmatics.

Of the 508 subjects who underwent a successful sputum induction, 211 (42%) had eosinophilic inflammation (≥3% eosinophils), 80 (16%) neutrophilic inflammation (≥76% neutrophils), 14 (3%) mixed granulocytic and 203 (40%) paucigranulocytic (sputum eosinophil count <3% and sputum neutrophil count <76%) inflammation (Tables 2 and 3, Figure 1). These proportions were rather similar after exclusion of steroid treated patients (Table 4). Compared to paucigranulocytic phenotype, eosinophilic, neutrophilic and mixed granulocytic phenotypes were characterised by a poorer lung function. Eosinophilic phenotype exhibited higher frequency of atopy, higher levels of IgE, higher bronchial hyperresponsiveness to methacholine, higher FE_NO_ levels and lower asthma control compared to paucigranulocytic. Mixed granulocytic phenotype had higher levels of fibrinogen, the lowest lung function and the highest degree of bronchial hyperresponsiveness to methacholine (Tables 2 and 3).

**Table 2:** Demographic characteristics according to the inflammatory phenotype

	**Paucigranulocytic phenotype**	**Eosinophilic phenotype**	**Neutrophilic phenotype**	**Mixed granulocytic phenotype**
N.	203 (40%)	211 (41.5%)	80 (15.7%)	14 (2.8%)
Sex (M/F)	72/131	101/110*	21/59	7/7
Age, yrs	51 (21–86)	51 (19–87)	57 (21–84)	68 (31–88)
Atopy (Y/N)	100/103 (49%)	140/71 (66%)*	48/32 (60%)	8/6 (57%)
Smoking (Y/N)	48/155 (23%)	38/173 (18%)	12/68 (15%)	3/11 (21%)
ICS therapy				
-Steroid naïve	66 (32.5%)	59 (28%)	25 (31.3%)	3 (21.4%)
-Low dose	31 (15.3%)	37 (17.5%)	4 (5%)*	1 (7.2%)
-Moderate dose	58 (28.6%)	54 (25.6%)	23 (28.7%)	3 (21.4%)
-High dose	48 (23.6%)	61 (28.9%)	28 (35%)	7 (50%)

*p < 0.05, **p < 0.001, ***p < 0.0001 *Paucigranulocytic asthma is used as the comparator.

*ICS*: inhaled corticosteroids. Low dose: ≤500 μg/day beclomethasone; Moderate dose: 500-1000 μg/day beclomethasone; High dose: >1000 μg/day beclomethasone.

**Table 3:** Functional and inflammatory characteristics according to the inflammatory phenotype

	**Paucigranulocytic phenotype**	**Eosinophilic phenotype**	**Neutrophilic phenotype**	**Mixed granulocytic phenotype**
IgE, kU/l	84 (1–7338)	211 (3–17183)***	107 (2–7338)	346 (1–2063)
Blood eosinophils,/mm^3^	160 (0–1220)	360 (0–3220)***	170 (20–1020)	420 (190–3040)***
Blood eosinophils,%	2 (0–13)	4.5 (0–26)***	1.9 (0.2-15)	5 (1.3-30)***
Blood neutrophils,/mm^3^	4030 (76–11080)	4220 (1820–15410)	5000 (2070–10440)	4245 (3520–6170)
Blood neutrophils,%	59 (27–82)	55 (32–91)*	62 (42–80)	59 (43–68)
FEV_1_,% predicted	90 ± 17	80 ± 20***	79 ± 20***	72 ± 14***
FEV_1_/FVC,%	77 ± 9	71 ± 10***	72 ± 11***	69 ± 9***
TLC,% predicted	99 ± 16	102 ± 18	102 ± 18	101 ± 14
FRC,% predicted	103 ± 27	104 ± 19	119 ± 32	111 ± 22
KCO,% predicted	90 ± 19	92 ± 21	91 ± 19	100 ± 10
PC_20_, mg/ml	4.42 (0.13-16)	2.02 (0.025-16)**	3.22 (0.05-16)	1.08 (0.53-2.2)**
Reversibility,%	8 ± 9	15 ± 17**	8 ± 10	12 ± 10
Sputum eosinophils,%	0.4 (0–2.9)	18 (3–94)	0.2 (0–2.8)	4.3 (3–8)
Sputum neutrophils,%	41 (0–76)	33 (0–76)	87 (77 – 100)	82 (76–92)
Fibrinogen, g/l	3.1 (2–6.3)	3.1 (2–7.2)	3.3 (1.9-10)	4.1 (2.7-6.3)*
CRP, mg/l	1.6 (0.2-14)	1.8 (0.2-14)	2.3 (0.2-10)	1.9 (1.1-6)
FE_NO_, ppb	16 (1–128)	53 (2–247)***	22 (0–192)	41 (12–161)*
ACQ	1.82 ± 1.15	2.16 ± 1.36*	2.09 ± 1.88	2.09 ± 1.16
Global AQLQ	4.6 ± 1.3	4.58 ± 1.34	4.76 ± 1.46	4.45 ± 1.74
-Emotion	4.92 ± 1.35	4.57 ± 1.63	4.9 ± 1.76	4.64 ± 1.83
-Symptoms	4.46 ± 1.46	4.42 ± 1.43	4.65 ± 1.54	4.39 ± 1.5
-Activity	4.71 ± 1.36	4.79 ± 1.44	4.84 ± 1.49	4.55 ± 1.80
-Environnement	4.48 ± 1.50	4.55 ± 1.55	4.81 ± 1.67	4.70 ± 2.15

*p < 0.05, **p < 0.001, ***p < 0.0001 *Paucigranulocytic asthma is used as the comparator.

Data are presented as mean ± SD or median (range); PC_20_ is expressed as geometric mean (range). *ACQ*, Asthma Control Questionnaire; *AQLQ*, Asthma Quality of Life Questionnaire; *FE*_*NO*_, exhaled nitric oxide; *FEV*_*1*_, forced expiratory volume in 1 s; *PC*_*20*_, concentration required to provoke a fall in FEV_1_ of 20% or more; *FVC*, forced vital capacity. *CRP*, C-Reactive Protein; *FRC*: Forced residual capacity; *KCO*: Carbon monoxide transfer coefficient; *TLC*: total lung capacity. Paucigranulocytic asthma is used as the comparator.

**Table 4:** Proportion of asthma inflammatory phenotypes in steroid naïve and steroid-treated patients

	**Paucigranulocytic phenotype**	**Eosinophilic phenotype**	**Neutrophilic phenotype**	**Mixed granulocytic phenotype**
**Steroid naïve (n = 153)**	65 (42.5%)	60 (39.2%)	25 (16.3%)	3 (2%)
**Steroid-treated (n = 355)**	138 (38%)	151 (43%)	55 (15%)	11 (3%)*

*p < 0.05.

**Figure 2:**
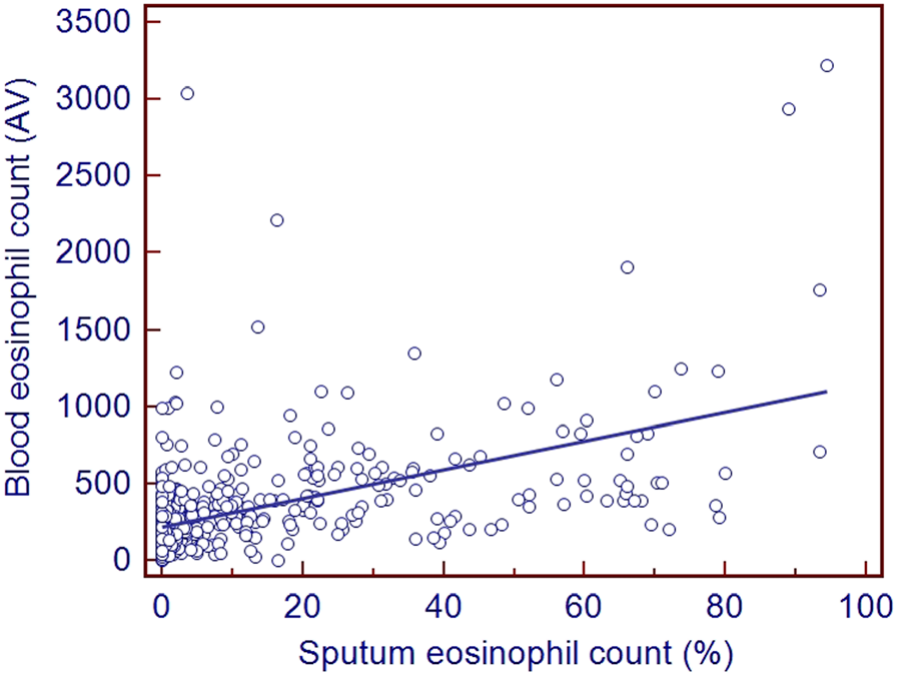
**Relationship between sputum eosinophil count and blood eosinophil count in a cohort of unselected patients with asthma (n = 508) by Spearman correlation.** There is a significant correlation between these two parameters (p < 0.0001, Rs = 0.6). AV: absolute value of blood eosinophils (/mm^3^).

**Figure 3:**
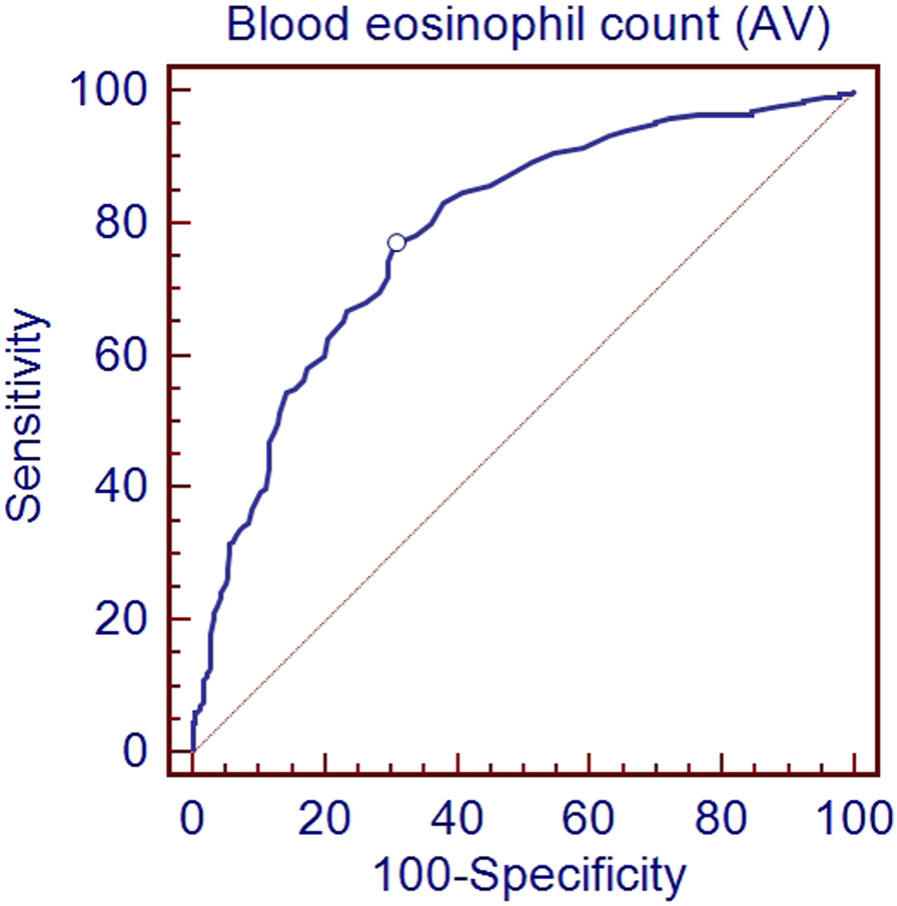
**Receiver-operating characteristic (ROC) curve for the whole group to determine the blood eosinophil count value that best identified a sputum eosinophilia ≥3%.** The optimum cut-off point was 220/mm^3^ (Sensitivity 77%, specificity: 70%, AUC: 0.790, p < 0.0001).

**Figure 4:**
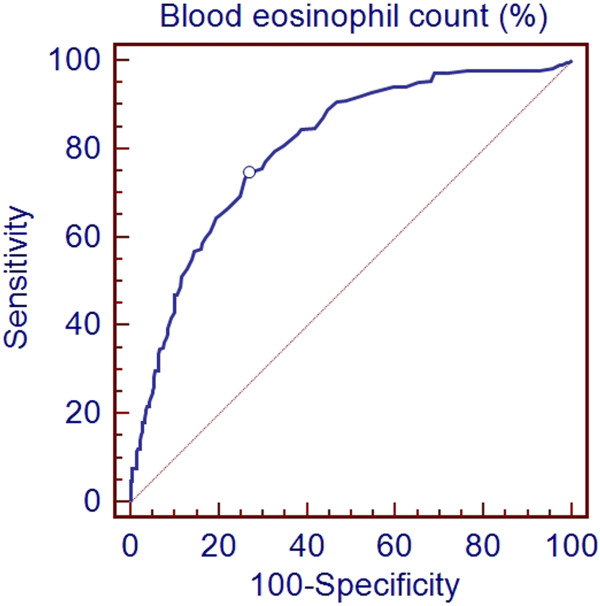
**Receiver-operating characteristic (ROC) curve to determine the blood eosinophil count percentage that best identified a sputum eosinophilia ≥3%.** The optimum cut-off point was 3% (Sensitivity: 75%, specificity: 73.4%, AUC: 0.81, p < 0.0001).

For the whole group there was a significant positive relationship between blood eosinophil count, either expressed as percentage or absolute value, and percentage of sputum eosinophil count (r = 0.6, p < 0.0001; r = 0.6, p < 0.0001; respectively; Figure 2). Using the ROC curve method we found that a blood eosinophil count ≥220/mm^3^ yielded 77% sensitivity and 70% specificity (Area under the curve (AUC) = 0.79, p < 0.0001, Figure 3) for identifying a sputum eosinophil count ≥3% in the whole population. By constructing ROC curve we found that a cut-off value of 3% for percentage of blood eosinophils was able to identify the presence of a sputum eosinophil count ≥3% with 75% sensitivity and 73% specificity (AUC = 0.81, p < 0.0001, Figure 4). The measure of blood eosinophils was as efficient as FE_NO_ (cut-off = 41 ppb, AUC 0.79, p < 0.0001) for identification of sputum eosinophilia ≥3%. The comparison of the AUC for both tests failed to demonstrate a significant difference (p = 0.77).

We further sought to determine the factors associated with sputum eosinophilic phenotype using multiple logistic regression models. When combining all variables into the model, percentage of blood eosinophils (logit-transformed; p < 0.0001), FEV_1_/FVC (p = 0.0021), FE_NO_ (log-transformed; p < 0.0001), and IgE (log-transformed; p = 0.0085) were independent factors associated with the presence of sputum eosinophilic inflammation (Table 5). Using those variables, we established a formula to predict the presence of a sputum eosinophil count ≥3%:

$$\begin{array}{l} \ln\left(\pi /\left(1-\pi \right)\right)=L=2.92+1.218\\ \;\times \ln\left(\left(\mathrm{Blood}\;\mathrm{eos}\%\right)/\left(100-\mathrm{Blood}\;\mathrm{eos}\%\right)\right)\\ \;+0.217\times \ln\left(\mathrm{IgE}\right)-0.039\\ \;\times \left(\mathrm{FEV}1/\mathrm{FVC}\%\right)+0.844\times \ln\left(\mathrm{FENO}\right) \end{array}$$

π: probability of sputum eosinophil count ≥ 3%.

Blood eos%: Blood eosinophil count in %.

To test the ability of the equation to predict sputum eosinophilia, we recruited a validation population of 138 asthmatics that underwent FE_NO50_, spirometry, sputum induction and gave a blood sample. The agreement between predicted and observed value of sputum eosinophil counts gave a Cohen Kappa’s coefficient of 0.59 (p < 0.0001, lower limit of confidence interval = 0.43). The specificity and sensitivity were 62.7% and 93.7% respectively while the PPV was 67% and the NPV was 93%.

**Table 5:** Independent predictors of sputum eosinophilia

**Parameter**	**β**	**SE**	**p-value**
**Logit Blood eosinophils,%**	1.218	0.19	P < 0.0001
**Ln IgE**	0.217	0.08	P = 0.0085
**FEV**_**1**_**/FVC,%**	−0.039	0.01	P = 0.0021
**FE**_**NO**_	0.844	0.16	P < 0.0001

*Ln*, Natural logarithm; *FEV1*, Forced expiratory volume in 1 s; *FVC*: Forced vital capacity; *FENO*, exhaled nitric oxide; SE: standard error.

As far as the sputum neutrophilic phenotype is concerned, there was a weak correlation between sputum and blood neutrophil count taken in percentage (r = 0.19, p = 0.0015) but not in absolute value (r = 0.19, p = 0.11). Using the ROC curve method, we found a cut-off of 4960/mm^3^ and 66% respectively giving a sensitivity of 49% and 37%, a specificity of 70% and 90%, p = 0.03 and p = 0.003, AUC = 0.59 and AUC = 0.63 respectively.

However, when combining all variables into the logistic model, age (p = 0.006) and Functional residual capacity (FRC, p = 0.001) were independent factors associated with sputum neutrophilia while blood neutrophils were not significant in this case. The formula to predict the presence of a sputum neutrophil count ≥ 76% was:

$$\ln\left(\frac{\pi }{1-\pi }\right)=L=-5.32+0.032\times \mathrm{age}+0.022\times \mathrm{FRC}$$

π: probability of sputum neutrophil count ≥ 76%.

To test the ability of the equation to predict sputum neutrophilia, we recruited a validation population of 178 asthmatics that underwent spirometry and sputum induction. The agreement between predicted and observed value of sputum neutrophil counts gave a Cohen Kappa’s coefficient of 0.24 (p < 0.0001, lower limit of confidence interval = 0.12). The specificity and sensitivity were 21% and 97% respectively**.** Patients receiving moderate to high dose ICS had higher sputum neutrophil count (47.3%) than patients receiving low dose ICS (38.8%, p = 0.017). Smokers did not have significantly higher proportion of neutrophils in their sputum (Median 48.9%) than ex-smokers (Median 50.6%, p = 0.68) or never smokers (Median 44%, p = 0.19). However, neither smoking status nor the dose of inhaled corticosteroids was able to predict elevated sputum neutrophil count.

## Discussion

In a large cohort of asthmatics encompassing all disease severity spectrum, eosinophilic and pauci-granulocytic were the most frequent phenotypes while neutrophilic asthma represented less than one fifth of the inflammatory patterns. Independent predictors of sputum eosinophil count ≥3% were the percentage of blood eosinophils, low FEV_1_/FVC, high FE_NO_ and IgE levels. A cut-off value of 220/mm^3^ or 3% for blood eosinophils performed equally to FE_NO50_ to identify the presence of a sputum eosinophil count ≥3%. Independent predictors of sputum neutrophilia were advanced age and high FRC while blood neutrophil count was not.

The proportion of asthmatics with raised sputum eosinophil counts was 46% in our series. Gibson et al. found that 41% of non-smoking asthmatics had a sputum eosinophil counts >2.5% [18]. The proportion of eosinophilic asthma reported by Louis [11] and Green [1] was higher but the thresholds used in those studies were 2 and 1.9% respectively. Our results are similar to the study of Simpson conducted on 93 subjects using thresholds of 1 and 61% for eosinophilic and neutrophilic inflammation respectively. This group found 41% eosinophilic asthma, 20% neutrophilic asthma, 31% paucigranulocytic asthma and 8% mixed granulocytic asthma [19]. The highest thresholds used in our study certainly explain the lower proportion of mixed granulocytic and neutrophilic asthma and the higher proportion of paucigranulocytic asthma in our patients. A very recent American multicentre study from McGrath et al. showed that paucigranulocytic asthma was the dominant phenotype accounting for more than 50% of patients while eosinophilic asthma (sputum eosinophil count ≥2%) represented roughly 25%. As in our study, the neutrophilic phenotype (sputum neutrophil count > 61%) was quite rare representing less than 15% of the patients while the mixed granulocytic phenotype was less than 5% [20].

Lung function was less altered in those patients with paucigranulocytic phenotype while eosinophilic phenotype exhibited higher FE_NO_ levels, higher proportion of males and atopic patients, higher bronchial hyperresponsiveness and lower asthma control. Those results are in accordance with previous studies [11,21-24]. Like Hastie et al. [25], we identified patients with mixed granulocytic sputum inflammation exhibiting the lowest lung function. Moreover, the mixed granulocytic phenotype had higher serum fibrinogen values pointing to systemic inflammation in this subgroup. This interesting finding has not been reported so far in asthma but raised fibrinogen levels have been demonstrated to be associated with reduced FEV_1_ and increased risk of Chronic Obstructive Pulmonary Disease (COPD) in a population study [26]. As for neutrophilic asthma there was no special characteristic that distinguishes this group from the other inflammatory patterns. In particular neutrophilic asthmatics did not display higher serum C-Reactive Protein and fibrinogen levels.

Among the factors shown to contribute to airway eosinophilia, blood eosinophils came first when performing a multiple logistic regression. There are few studies assessing the ability of blood eosinophils to identify airway eosinophilic inflammation. Previous studies showed that peripheral blood eosinophil count was correlated with bronchoalveolar lavage eosinophil count [27] and sputum eosinophil count [28-30]. These studies have, however, investigated a limited number of asthmatics and did not provide any threshold value of blood eosinophils as marker of airway eosinophilia. The recent American multicentre study found a threshold value of 220/mm^3^ as the best compromise for predicting sputum eosinophil count ≥2% [20]. Another recent study conducted in COPD showed that a cutoff of 2% peripheral blood eosinophils had a sensitivity of 90% and specificity of 60% for identifying a sputum eosinophilia of greater than 3% at exacerbation. In our study, we confirmed the correlation between blood and sputum eosinophilic inflammation in a large cohort of patients. We found the best threshold being 220/mm^3^ and 3% and these thresholds being as effective as FE_NO_ at predicting uncontrolled airway eosinophilic inflammation. Our findings are in keeping with those recently reported by McGrath et al. [20]. Compared to blood eosinophils, FE_NO_ has however the advantage of giving immediate results and its measurement is more comfortable to the patient. In this study the performance (threshold, sensitivity, specificity) of FE_NO_ to identify sputum eosinophil count is very similar to the one we reported previously [10]. The fact that FE_NO_ and blood eosinophil counts came out as independent predictors of sputum eosinophilia in the multiple logistic regression suggests that these two markers reflect different mechanisms promoting the recruitment of eosinophils into the airways.

Other independent factors to be shown associated with prominent sputum eosinophilia are FEV_1_/FVC and total serum IgE level. Previous studies have shown that FEV_1_/FVC, an index of airway narrowing, was correlated to sputum eosinophilia [31] and eosinophilic asthma has been recognized to be frequently associated with atopic disease [1,7]. In our study, total serum IgE levels were however best predictor of sputum eosinophilia than atopy per se, in line with a recent study [20]. From the biological properties of IgE it can be speculated that high tissue IgE may prime local mast cells and activate them even without intervention of allergens [32]. In this view it is interesting to note that airway mast cell activation demonstrated by tryptase release is a phenomenon found to be associated with sputum eosinophils in asthma [33] and COPD [34].

The same approach as for eosinophils was used to predict the presence of sputum neutrophils. Despite statistically significant correlation between sputum and percentage blood neutrophils, the strength of the relationship was rather poor. As opposed to what we found with eosinophils, multiple logistic regression analysis demonstrated the inability of blood neutrophils to predict uncontrolled sputum neutrophilic inflammation. The accumulation of airway neutrophils has been reported to be directly associated with the activation state of circulating neutrophils in response to the chemokine Interleukin 8 [35,36]. Baines et al. found a correlation between plasma neutrophil elastase and neutrophilic airway inflammation [37]. Those data suggest that airway neutrophilic accumulation could be due to an enhanced neutrophil activation and migration to the airways independently of the number of circulating cells. Moreover, it has been shown that neutrophils can be retained in the pulmonary microvasculature due to their low deformability, resulting in a higher concentration than in the systemic circulation. It is thought that this high concentration of the cells facilitates their effective recruitment to sites of inflammation [38]. It seems likely that many could leave the circulation by chemoattraction, entering the lung without necessarily having a detectable effect on circulating levels.

From a multiple logistic regression two factors came out as being independently associated with sputum neutrophilia. In keeping to what was found by Thomas et al. in healthy subjects [17] and Woodruff et al. [23] in asthmatics, age appeared to be a critical factor in our cohort with sputum neutrophilia rising with age. In addition to age we also found that FRC was an independent factor. This suggests that airway neutrophils may contribute to reduction of inspiratory capacity seen in some asthmatics. Accordingly, two pediatric studies reported that percentage neutrophils in bronchoalveolar lavage directly correlated with air trapping (FRC) in children with cystic fibrosis [39,40]. However, neither smoking status nor the dose of inhaled corticosteroids was able to predict the presence of sputum neutrophil count.

## Conclusion

This study shows that eosinophilic and pauci-granulocytic are the most frequent asthma phenotypes in a large unselected asthmatic population. Like FE_NO_, blood eosinophil counts may provide a practical alternative to predict sputum eosinophilia ≥3%. In contrast, sputum neutrophilia is only poorly related to blood neutrophil count.

## Competing interests

The authors declare that they have no competing interests.

## Authors’ contributions

Conception and design of the study: FS, RL; Data collection: FS, MM, JS, MH, RL; Analysis and interpretation: FS, RL, LS; Drafting the manuscript for important intellectual content: FS, RL, LS. All authors read and approved the final manuscript.

## Pre-publication history

The pre-publication history for this paper can be accessed here:

http://www.biomedcentral.com/1471-2466/13/11/prepub
